# Acute pain sign recognition by dog owners in a home setting

**DOI:** 10.1371/journal.pone.0345418

**Published:** 2026-04-15

**Authors:** Silvia Gardeweg, Chiara Valtolina, Abraham Calero Rodriguez, Ineke van Herwijnen

**Affiliations:** 1 Division Animals in Science and Society, Department Population Health Sciences, Faculty Veterinary Science, Utrecht University, Utrecht, Netherlands; 2 Department Clinical Sciences, Faculty Veterinary Science, Utrecht University, Utrecht, Netherlands; Lyon College, UNITED STATES OF AMERICA

## Abstract

To identify behavioural changes indicative for acute pain in dogs that are recognized by their owners and the wording used to describe these, we asked owners of 51 dogs treated at the Small Animal Clinic of Utrecht University, to document and video tape observed behavioural changes and estimate their dog’s level of pain within the first week after clinical discharge. Quantitative data was tested with logistic regression analysis for the predictability of certain behavioural changes to occur at higher owner-estimated pain scores. Owner-recorded videos were analysed independently by three veterinarians to provide a professional reference perspective. Free text entries from participants were analysed qualitatively and the wording used by owners whose dogs were deemed in pain according to the veterinary video evaluation, was extracted. We found that the most often reported behavioural changes on the day of discharge, and on the two days thereafter were: changes in walking (72.1% *n* = 44/61), playing with an object (70.5%, *n* = 43/61) and playing with the owner (68.9%, *n* = 42/61). The empirical data indicated a decrease in, e.g., playing behaviour, explorative behaviour and eating. Logistic regression analyses showed a significant association between owner-estimated pain scores and behavioural changes for these tested items. No correlation was found between pain estimation by the veterinarians and the dog owners. The qualitative text analysis of entries from owners whose dogs were deemed in pain, provided insight into wording used to describe pain-related behavioural changes by owners, that may be used in the context of developing an owner-directed acute pain scoring instrument. Such an instrument is needed to help owners recognize and correctly interpret pain-induced behavioural changes, to ensure adequate pain management and animal welfare after clinical discharge.

## Introduction

Pain is a multidimensional phenomenon including sensory, affective and cognitive components [1,2]. While acute pain is an evolutionary preserved experience, serving to protect animals from potential threats, chronic pain results from pathological changes within the pain transmission system, and persists longer than its initial cause, hence serving no biological purpose [3]. The transition from acute to chronic pain used to be defined primarily in terms of time, until other factors, e.g., the emotional status, were seen to contribute to this mechanism [4]. Despite its important function, acute pain represents a source of suffering in the perioperative setting, with a study in humans revealing 66% of patients reporting moderate, severe or extreme pain immediately after surgery and 59% within the first two weeks after surgery [5]. Inadequate treatment of acute pain promoted pathophysiological changes and the development of maladaptive behaviours [6], potentially leading to long term pain states and pain-related disability [7]. Described complications occur even in humans capable of verbal communication, thus in species able to communicate about opportunities for adequate pain mitigation [8]. Non-human animals may not experience such communication advantages and even benefit from concealing pain as to not come across vulnerable to, e.g., predators [9]. Consequently, interspecies differences in pain expression exist and this may hamper dog pain recognition by their owners [10,11].

With comprehensive pain assessment forming the basis for effective pain treatment [12], several methods for an improved identification of pain signs in different species have been developed, predominantly focusing on species-specific behavioural changes and facial expression [13]. Similarities in pain-induced facial expression changes have been identified in a variety of species and are utilized for animal welfare assessment in different settings [14–17]. While for cats, an automated pain recognition tool has been developed for supporting owner-directed pain assessment [18], automated emotion recognition in dogs remains complicated by the huge visual variety among dog breeds [19].

The veterinary profession uses so-called pain scales to overcome the difficulties in pain assessment in dogs and to ensure effectiveness of analgesic treatment [20,21]. These pain scales offer a standardised set of pain evaluation criteria and allow for monitoring of the individual animal’s pain experience [20,21]. An example of such a pain scale regards the validated short form of the Glasgow composite measure pain scale (CMPS-SF), a questionnaire containing 30 descriptors divided into 6 categories. Descriptors are numerically ranked according to represented pain severity and the assessor chooses the descriptor best fitting the respective dog’s behaviour [20]. For the purpose of acute pain assessment, the CMPS-SF refers to situational behavioural changes, whereas chronic pain scales focus on gradual developments and changes in daily routine. Recognizing the important role of the owner in assessing their dog’s pain, pain scales have been developed in recent years to help animal owners document and assess their dog’s chronic pain [22–24]. Yet, for acute pain, such pain scales are presently available for professional use in a clinical situation, requiring a trained and skilled observer [25]. Interviews with owners of dogs suffering from painful conditions, suggest that pain-related behavioural changes are recognized by untrained observers, but are at risk of being contextualized [26]. Insecurity in interpretation of behavioural changes as pain signs is as well reflected in the majority of owners focusing on movement-based changes when assessing their dog’s pain [27]. However, incorporating the finding that pain is not only a sensory but also an emotional experience, a recent study using free choice profiling (FCP) methodology revealed that owners had a good ability to identify the expression of pain in dogs, including a broader spectrum of pain signs than movement-based changes. FCP is a qualitative method, allowing for use of own vocabulary [28].

Considering above-described study results, a predefined list of behavioural changes indicative for pain in dogs, presented in wording familiar to owners, may facilitate a dog’s acute pain assessment in the home environment. In this study we aimed to: (1) identify pain-related behavioural changes recognized by owners after clinical discharge of their dog and the vocabulary they use to describe these changes, (2) assess for those dogs deemed in pain by veterinarians which behavioural changes dog owners indicate and which vocabulary they use to indicate behavioural changes, and (3) integrate owner vocabulary, professional observations, and validated pain scale items to create a preliminary framework for a future owner-directed pain assessment instrument.

## Materials and methods

### Ethical statement

The Animal Welfare Body of the University of Utrecht declared our study, based on owner-directed monitoring of the dogs, to not constitute an animal experiment. In addition, approval was sought and obtained from the Science-Geosciences Ethics Review Board of Utrecht University. All invited dog owners were provided with written information about the study aim and asked for consent by digitally indicating their consent and willingness to partake, before accessing the online questionnaire. Participants were given the opportunity to withdraw from the study at any time. No minors were included in this study. As no previous studies exist for animal caretakers in the home environment, reporting on their dog’s pain signs, power analysis could not validly be made for our project. As an alternative, we assessed studies with a resemblance in method, topic and research questions and based our minimal number on these studies; such as of Sparks et al., 2018 [29].

### Data collection

#### Participant recruitment.

Owners of dogs undergoing surgical or medical treatment requiring hospitalization at the Department of Clinical Science of Companion Animals, Utrecht University, hereafter ‘Faculty Clinic’, were invited to participate in the study via e-mail. Dogs of all breeds and ages were allowed to take part, regardless of previous or co-existing conditions and the kind of treatment they underwent. The impact of medical and behavioural experience of owners in the study context was communicated, and two potential candidates withdrew from participation as these were veterinary professionals.

#### Online questionnaire and videos.

Potential participants received a link to Qualtrics survey software (Qualtrics, Provo, UT) platform and after digitally indicating to have received information about the study’s purpose and conditions and agreeing with these, were given access to the questionnaire. The questionnaire was available online between June 13^th^ and November 20^th^ 2024 in Dutch and incorporated the results of a literature review regarding pain-induced behavioural changes and clinical experience regarding hospitalization-associated behavioural changes. The questionnaire allowed for generation of quantitative and qualitative data and was pretested with two native Dutch speakers. Intending to identify dogs’ behavioural changes recognized by their owners, the questionnaire started with open questions regarding the dogs’ general behaviour, posture and facial expression. Subsequently, participants were asked to indicate changes in the behavioural categories of walking, sleeping, eating, urination, playing with the owner and with an object, interaction through petting, greeting, staying close to the owner and exploration of the environment. For each behaviour perceived as altered, owners were asked to give a description of their observations in a free text field. Next, two questions asked directly the owners’ opinions on their dog’s pain experience. The first question regarded the likelihood of a dog experiencing pain (1 = not at all likely, 2 = not likely, 3 = don’t know, 4 = likely, 5 = very likely). The second question rated the intensity of the dog’s possible pain experience with an 11-point Likert scale (0 = no pain, to 10 = the worst pain imaginable) [30]. The questionnaire ended with two open questions regarding specific signs that were telling the owner whether their dog was in pain or not (see S1 File for all questions in English). Owners were asked to complete the questionnaire four times: on the first, second, third and seventh day after discharge from the clinic. Additionally, they were asked to upload videos of their dogs displaying behavioural changes they believed to be pain-associated on each day of documentation (video instructions are available in S1 File). Only completed questionnaires were used in our study, as we deemed all answers unmissable to allow for qualitative data analyses, e.g., the answers regarding how the owner perceived the dog’s pain experience.

### Analysis

#### Veterinary video analysis.

Owner-submitted videos were individually evaluated for the respective dogs’ level of pain by three veterinarian assessors (a veterinarian with in-house patient care-focused experience, a European diplomate in Veterinary Anaesthesia & Analgesia (ECVAA) and a European diplomate of Veterinarian Emergency and Critical Care (ECVECC). Considering that the participating dogs were not examined personally by the authors, this assessment served as a professional reference regarding the dogs’ level of pain, in addition to the owners’ assessments. The three veterinarians were unaware of the specific treatments the dogs underwent, on which day after clinical discharge videos were recorded, which pain medications the dogs received and their owners’ pain scoring results. None of the participating dogs underwent current or past treatment by one of the veterinarians assessing the videos. All assessors were asked to evaluate the videos alone, watch the videos as many times as they deemed necessary and make use of the slow-motion function when needed. Assessment results were documented in a Microsoft Excel (Microsoft corporation, Microsoft 365 Subscription) table, providing one column for pain scoring on an eleven-point Likert scale (with 0 representing no pain and 10 representing the worst pain imaginable), identical to the pain scale provided within the online survey to participating dog owners. A second column allowed for describing the behaviours that led to the scoring result. A third column allowed for indicating if analgesic intervention would be deemed necessary, based on video observation. A pain score > 5 was used as threshold to define the presence of moderate to severe pain. After completion of all video assessments, the evaluations were discussed between all authors and it was agreed to categorize each dog, that was evaluated to experience pain by at least one of the assessors, to be in pain.

#### Quantitative statistical analysis.

Despite the proposed fixed weekdays, participants provided documentation scattered from the day of hospital discharge to the 8th day thereafter. Hence, three time frames were defined: a first time frame (day of clinical discharge, first and second day thereafter), a second time frame (day three, four and five after discharge) and a third time frame (day six, seven and eight after discharge). Questionnaire data were transferred from Qualtrics survey software (Qualtrics, Provo, UT) to Microsoft Excel (Microsoft corporation, Microsoft 365 Subscription) and IBM SPSS Statistics for Windows (IBM, Version 29.0). For each of these time frames, numerical and qualitative data extracted from questionnaire entries were analysed. The numerical data regarded owner-reported behavioural changes for each of the behavioural categories questioned in the online survey. These were tested separately as a dependent variable and dog pain scores as the independent variable in logistic regression analyses, regarding values of p < 0.005 as significant, after Bonferroni correction to the P-values, by dividing a p-value of 0.05 by the number of 10 tests done, regarding values of p < 0.5 as a trend, and presenting odds ratios (OR), confidence intervals (CI), coefficient b, standard error (S.E.), Z-scores and p-values. We present exact p-values for p-values ≥ 0.001, and as p < 0.001 for p-values below <0.001 for readability. For the numerical data derived from the veterinary video analysis, namely the pain scoring results, means ± standard deviations (S.D.) are presented for the scores per assessor and per all three assessors. Pearson correlations between the assessors were determined, regarding p-values < 0.05 as significant and we have indicated the interpretation of the magnitude of the correlation coefficients following Schober et al. (2018) [31]. Cronbach’s alpha assessed internal consistency of the scoring method. These calculations were done for: a) all videos grouped together, b) videos recorded during the first time frame only and c) videos of dogs that underwent orthopaedic surgery and soft tissue surgery or medical treatment separately.

#### Qualitative analysis.

Qualitative data analysis has increasingly been used in research during the past years [32,33], especially for the purpose of investigating subjective states, such as emotions [34,35]. In the process of qualitative text analysis, the text is coded and categorized. This was traditionally done manually, by colouring text parts with pens, then cutting and sorting them. These tasks are today taken over by software such as NVivo, helping to maximize efficiency [36]. In our study, we used inductive content analysis (ICA), to analyse owner-provided data [37]. Inductive content analysis is a specific method for qualitative text analysis. Owners’ descriptions of dog pain signs provided through open text answer options, were translated from Dutch into English by a native Dutch speaker and subsequently transferred into NVivo software (Lumivero, version 1.5.2) for coding purposes. The coding was done by one researcher. The words and phrases were labelled (coded) as they emerged from the text, thus inductively. This approach enabled to distil codes emerging from participants’ own words [38,39]. The codes were then transferred from NVivo software to Microsoft Excel, after which a second researcher checked whether this transferring had not resulted in errors. Considering that the coding was conducted by one researcher, interrater reliability was not assessed. Next, these codes were grouped into logical categories of owner descriptions of their dog’s pain-related behavioural changes, recognized and documented by owners after their dog’s clinical discharge. Codes and categories were kept that were entered by the owners whose dogs were deemed painful according to the veterinary video analysis. These remaining codes reflected how dog owners describe pain-related behavioural changes. The codes and categories were discussed by a group of researchers, including the researcher who coded the data and the veterinarians who assessed the dog’s videos for pain levels. Coding/categorisation that needed to be added for completeness and on which this group of researchers unanimously agreed, was added and we report in a supplementary file (S2 Table) the source of each of the codes and categories. A final source was deemed necessary as none of the dogs had been evaluated to be in unbearable pain during the video analysis or described in such terms by their owners. With our long-term aim of creating an acute pain scale for dogs in the home environment, we therefore added behavioural items corresponding to extreme high levels of pain, which were extracted from the short form of the Glasgow Composite pain scale CMPS-SF [20]. We compared our findings with the literature on development and validation of pain scales used by the veterinary profession [20,40–42].

## Results

### Participants and dog’s characteristics

From 398 owners of dogs being invited to take part in the study, 51 participated, corresponding to a response rate of 12.8%. Age of the respective dogs ranged from 4 months to 12 years, with 15 dogs being < 2 years, 15 dogs 2–6 years and 21 dogs being ≥ 7 years old. The distribution between male and female dogs was nearly equal, as shown in S1 Table, with all details provided in Table 1. Treatment of the dogs included 29 soft tissue surgeries, 2 medical treatments and 20 orthopaedic surgeries. The analgesic medication dogs were sent home with, included non-steroidal anti-inflammatory drugs (NSAIDs) in 36 cases, acetaminophen (paracetamol) in 22 cases and a gabapentinoid (gabapentin) in 23 cases, with 23 dogs receiving combinations of two or more analgesics. Twenty-eight of the participating dogs were discharged on the day they underwent surgery and 12 one day thereafter.

**Table 1:** Dog, treatment and analgesic characteristics.

Gender	Age (years, or months indicated with ‘m’)	Treatment/ Condition	Medication
f	8m	Eversion third eyelid	Chloramphenicol eye cream, Meloxicam
mc	10	Adenocarcinoma removal	Paracetamol
m	4	Hypophysectomy	Paracetamol
m	1	Intra hepatic portosystemic shunt	Paracetamol
fc	8	Arthrotomy	Paracetamol, Gabapentin
mc	12	Tumour excision	Carprofen
mc	4	Paw amputation	Carprofen, Gabapentin, Paracetamol
m	3	Castration	Carprofen
fc	6	Castration	Carprofen, Gabapentin
fc	9	Removal of multiple skin tumours	Paracetamol, Carprofen
fc	2	Patella luxation	Gabapentin, Meloxicam
m	1	Castration	Carprofen
mc	1	Castration	Meloxicam
mc	5	Excision interdigital mass	Carprofen
m	7	Urethrostomy	Carprofen
fc	7	Pancreatectomy	Paracetamol
f	5m	Ectopic ureter	Meloxicam
m	10	Tumour excision	Gabapentin, Paracetamol
fc	10	Tumour resection thoracic wall	Carprofen, Gabapentin, Paracetamol
fc	12	Patella luxation	Gabapentin, Meloxicam
f	1	Trichiasis	Carprofen
fc	7	Toe amputation	Meloxicam
f	2	Ovariohysterectomy	Meloxicam
f	1	Hip replacement	Gabapentin, Paracetamol
fc	2	Gastroduodenoscopy	Prednisolone
mc	9	Cataract removal	Prednisolone
fc	7	TPLO	Carprofen, Gabapentin, Paracetamol
fc	10	Mastectomy	Gabapentin, Meloxicam, Paracetamol
fc	11	Enucleation	Carprofen
m	7	Hip replacement	Carprofen, Gabapentin
f	5	Entropion, tumour removal nose	Carprofen, Gabapentin, Paracetamol
m	2	Hip replacement	Carprofen, Gabapentin
m	8	Hip replacement	Paracetamol
m	1	Hip replacement	Carprofen, Gabapentin
m	4	Revision Hip replacement	Carprofen, Gabapentin
mc	8	Hemiabdomen	Paracetamol
f	1	Laparoscopic ovariohysterectomy	Carprofen
mc	11	TPLO	Carprofen, Gabapentin
f	9m	Hip replacement	Carprofen, Gabapentin
m	1	Castration	Carprofen
fc	3	Foreign body	Paracetamol
m	4m	Pneumonia	Meloxicam
fc	7	Arthrotomy elbow joint	Carprofen, Gabapentin
m	1	Explorative laparotomy	Paracetamol
f	1	Ovariohysterectomy, umbilical hernia	Carprofen, Paracetamol
m	4	Patella luxation	Meloxicam
m	8	Paw amputation	Carprofen, Paracetamol
f	4m	Patella luxation bilateral	Meloxicam, Paracetamol
m	8	Adrenalectomy	Paracetamol
mc	6	Resection Osteosarcoma	Carprofen, Gabapentin
m	6	Skin resection planum nasale	Carprofen, Gabapentin

Characteristics of all dogs included (m = male intact, mc = male castrated, f = female intact, fc = female castrated).

A total number of 74 video recordings, referring to 22 dogs, was received. Video recordings had a mean ± S.D. duration of 51 ± 0.4 (2–150) seconds. 14 of the videos referred to dogs that were scored by their owners to have a high pain score (> 5) and 30 videos to dogs that were evaluated to have a low pain score (< 5; *n* = 8 for pain score of 5; 22 videos referred to dogs, whose owners did not know how to rate or did not provide a pain score). Mean ± SD pain intensity score, calculated from all owner-estimated pain scores, was 3.4 ± 2.7 (0–8; *n* = 51).

### Owner-reported dog acute pain experience and behavioural changes

Owner reporting on their dog’s acute pain resulted in *n* = 134 questionnaire returns, Of these, *n* = 31 were incomplete and excluded from further analysis. From the remaining *n* = 103 questionnaires, *n* = 61 referred to the day of discharge or to the first or second day thereafter. Behavioural changes registered by owners in this time frame were: changes in walking (72.1% *n* = 44/61), playing with an object (70.5%, *n* = 43/61), playing with the owner (68.9%, *n* = 42/61), urination (65.6%, *n* = 40/61), explorative behaviour (62.3%, *n* = 38/61), sleeping (60.7%, *n* = 37/61) and eating (49.2%, *n* = 30/61; *n* = 2 missing values). In addition to the quantitative indication of behaviours being seen as changed or unchanged, participant-provided free text entries. These free text entries indicated a decrease in playing behaviour, either with an object or the owner; as well as in eating and explorative behaviour, such as sniffing and wandering around. In contrast, frequency and length of urination and total length of sleeping periods were perceived as increased. Including all questionnaire entries, owners scored a mean ± S.D. pain score of 3.6 ± 2.6.

Logistic regression analysis revealed the owner estimated pain score to predict a change in the dog’s behaviour for interacting with the owner by petting, with a trend for the other behavioural items, except for urination (Table 2). The impact of the pain score was expressed in the odds ratio (OR), defining the probability of a behaviour being changed if the pain score increased with one point. One point increase in pain score elevated the chance of behavioural change for walking by 1.5 (OR = 1.5, confidence interval (CI) = 1.1–1.9), interaction in form of being petted by the owner by 1.4 (OR = 1.4, CI = 1.1–1.7), greeting the owner by 1.3 (OR = 1.3, CI = 1.1–1.6), exploring by 1.3 (OR = 1.3, CI = 1.1–1.7) and playing with the owner by 1.3 (OR = 1.3, CI = 1.1–1.6).

**Table 2:** Relationship between higher pain scores and the owner indicated behavioural change.

Variable	Coefficient b	S.E.	Z-score	p-value	OR	95% CI	Behaviour changed (% of all participating dogs)	Behaviour changed (% of dogs with owner pain score ≤ 5
Walking	0.37	0.14	2.72	0.006	1.45	1.11–1.90	63.93	76.47
Interacting with the owner by petting	0.32	0.11	2.87	**0.004**	1.37	1.11–1.70	27.9	47.06
Interacting with the owner by greeting	0.29	0.10	2.79	0.005	1.34	1.09–1.64	39.34	64.71
Exploring	0.29	0.11	2.78	0.005	1.34	1.09–1.65	59.02	76.47
Playing with the owner	0.26	0.11	2.38	0.017	1.29	1.05–1.60	54.1	64.71
Eating	0.25	0.10	2.47	0.014	1.28	1.05–1.57	47.54	58.82
Sleeping	0.23	0.10	2.18	0.029	1.25	1.02–1.54	52.5	64.71
Interacting with the owner by being close	0.21	0.10	2.17	0.030	1.24	1.02–1.50	50.81	82.35
Playing with an object	0.21	0.11	2.00	0.046	1.23	1.00–1.52	63.93	76.47
Urinating	0.16	0.10	1.68	0.093	1.18	0.97–1.42	52.5	52.94

Relationship between dog owner-documented behavioural changes and dog owner-provided pain scoring as divided in lower (<5) and higher (≥5) pain scores.

Logistic regression analysis for *n* = 103 questionnaire-entries indicates how owner-reported behaviour changed at higher pain scores given to dogs (p < 0.005 as significant, p < 0.05 as a trend).

### Veterinarian assessment of owner-submitted post treatment dog videos in the home environment

An independent subjective evaluation of the owner-recorded videos by three veterinarians resulted in mean pain scores of 3.9 ± 1.8, 4.2 ± 2.3 and 4.6 ± 2.9, respectively. Thus, all mean pain scores were above the mean pain score of 3.4 ± 2.7, reported by the animal owners. The pain scoring results of the Dipl. ECVECC and the general veterinarian did not correlate significantly with the pain scoring results of the dog owners, whereas the pain scoring results of the Dipl. ECVAA did correlate weakly (r = 0.35) but significantly (p = 0.02) with the pain scoring results of the dog owners.

The owner-estimated and veterinary pain scores were assessed separately for the first time frame defined, thus the first three days after clinical discharge. For this time frame the mean owner-estimated score was 4.3 ± 2.6 (0–8; *n* = 39) and the veterinarians’ scores 4.3 ± 1.9 (1–9; *n* = 44), 5.2 ± 3.0 (0–9; *n* = 44) as well as 4.3 ± 2.4 (0–9; *n* = 44). Veterinarians’ scores correlated weakly with owner scores (r = 0, r = 0.1 and r = 0.3, all p > 0.05); see S3 Table for all correlations.

Subsequently, the pain scores were assessed separately for dogs undergoing medical treatment or soft tissue surgery, and orthopaedic surgery. For dogs that underwent orthopaedic surgery, the mean owner-provided score was 4.1 ± 2.8 (0–7; n = 16), while the veterinarians scored: 4.3 ± 2.2 (1–9; *n* = 33), 5.8 ± 2.4 (0–9; *n* = 33) and 4.3 ± 1.5 (1–7; *n* = 3), respectively. Here also, veterinarians’ scores correlated weakly with owner scores (4.1 ± 2.8, 0–7, n = 16; r = 0, r = 0.1 and r = 0.2, all p > 0.5).

For soft tissue surgery and medical treatment, the mean scores were lower: owners estimated the level of pain on average with 2.8 ± 2.7 (0−8, n = 32) and the veterinarians with 3.7 ± 2.01 (1−9; *n* = 39), 4.2 ± 2.7 (0−8; *n* = 39) and 3.8 ± 3.1 (0−8; *n* = 39); with the individual veterinarians’ scores correlating moderately with r = 0.6, r = 0.7 and r = 0.7 (p < 0.001). Veterinarians’ scores again correlated weakly with owner scores (2.8 ± 2.7, 0−8, *n* = 32; r = −0.2 (p < 0.001), r = 0.1 (p = 0.001) and r = 0.3 (p = 0.10).

Cronbach’s alpha, reflecting the internal consistency of the evaluation method, was moderate (0.7) when the animal owner’s pain estimation was included in the analysis and high (0.8), when including the results of the veterinary video analysis only.

### Potential effect of gabapentin on behaviour

Of the participating dogs, 23 received gabapentin as an analgesic, which is known to cause sedation as a side-effect. A potential effect of gabapentin medication on the evaluation of the dog’s pain level was examined using logistic regression analysis (details are provided in S2 File). The European diplomate in Veterinary Anesthesia & Analgesia (ECVAA) was significantly (p = 0.048) more likely (OR= 2.67) to score pain with 5 out of 10 or higher in dogs that received gabapentin and slightly less likely (OR = 0.38) to apply a pain score lower than 5. A similar result was revealed for the European diplomate of Veterinarian Emergency and Critical Care (ECVECC); in this case the chance that a pain score of 5 or higher was applied increased more than four times (p = 0.007, OR = 4.29) when the dog had received gabapentin as an analgesic; the chance for scoring pain lower than 5 was slightly decreased in dogs treated with gabapentin (p = 0.003, OR = 0.19). No significant effects of the dog having received gabapentin as an analgesic was found on the pain scoring results of the non-specialized veterinarian and the dog owners.

### Qualitative analysis of free text questionnaire entries and veterinarians’ descriptions

Qualitative text analysis of participant-provided questionnaire entries using NVivo software (Lumivero, version 1.5.2), following an inductive approach, resulted in 104 codes, that were grouped into 18 categories. Subsequently, codes referring to wording of owners whose dogs were evaluated to be in pain according to the veterinary video assessment, were compared to those referring to the terms used by owners of dogs that had been evaluated to not be in pain. This approach was chosen to identify the wording used by owners to describe behavioural changes displayed by dogs that were considered to suffer from pain by veterinarians. From the codes that were predominantly or exclusively used by owners whose dogs were deemed to be in pain, 17 codes were dropped for being considered to not directly correlate with a dog’s pain experience but merely being associated with the previous hospitalisation. For example, many owners indicated changes in their dog’s urine colour shortly after clinical discharge, which could result from intravenous fluid administration [43]. Codes most often referring to the description of pain-related behavioural changes in dogs, were those categorised as ‘quiet and depressed’ (15.9%), ‘affectionate’ (11.9%), ‘movement reluctance’ (9.8%), ‘movement lacking’ (9.5%), ‘position and resting difficulty’ (8.5%). Table 3 provides the categories, codes assigned to each category and the frequency at which codes were mentioned by dog owners as a percentage of total codes (*n* = 378). Written descriptions provided by the veterinarians during their video assessment, were as well analysed qualitatively and terms leading to an improved description of pain-induced behavioural changes, were extracted. These were ‘lame’ (13.1%, *n* = 27/207), ‘lip licking’ (12.4%, *n* = 13/105), ‘uncomfortable position’ (9.8%, *n* = 10/102), ‘unnatural posture’ (7.7%, *n* = 5/65) and ‘reluctance to move’ (7.5%, *n* = 11/142). Ultimately, terms were adopted from the GCMPS, that describe behavioural changes attributable to high levels of pain: ‘growl’, ‘guard wounded area’, ‘increased respiratory rate’, ‘screaming’ and ‘rubbing of wound area’.

**Table 3:** Categorisation of owner-mentioned pain signs.

Category	Codes	Frequency of codes as % of total (*n*)
Aggression	Aggression to dogs and/or people, snapping	1.32% (5)
Alertness and stress	Alert, confused, ear movement, looking around, stressed	4.50% (17)
Elimination change	Less elimination, stool in small amounts, urinating less, house soiling	2.91% (11)
Fear and insecurity	Fearful after movement, frightened, insecure, panicking, shaking	3.70% (14)
Intake change	Drinking less, drinking more, eating less, eating with owner	3.97% (15)
Mobility limited	Careful, gets up with difficultly, lying down frequently, impaired moving, moving slowly, pausing, reluctance to walk, walking slowly	9.79% (37)
Movement impairment	Dragging a leg, unsteady gait, lame, lifting wounded leg	2.12% (8)
Movement lacking	No greeting, no jumping, no playing, no walking, not getting up, not moving, sitting still	9.52% (36)
Muscle tension	Hollow cheeks, rigid	0.79% (3)
Normal routine	Acting normal if active, normal routines	0.53% (2)
Owner avoidance	Avoid being touched, keeping distance	3.17% (12)
Owner directed	Alert when owner moves, asks for petting, attachment increased, attention seeking, interacting, interaction changed, looking at owner, staying close	11.90% (45)
Owner greeting	greeting enthusiastically, responding enthusiastically, tail wag	2.12% (8)
Position and resting difficulty	Changing position, getting up more often, hunched back, light sleep, restless, restless at night, sleepless, instable lying position, uncomfortable position, unnatural posture	8.47% (32)
Quiet and depressed	Calm, depressed, dull, indifferent, lethargic, lying head down, no reaction, quiet, sad eyes, walking head down	15.87% (60)
Sleepiness and tiredness	Sleeping a lot, sleepy, tired	7.41% (28)
Vocalisation change or absence	No vocalisation, vocalising, whining	2.12% (8)
Wound attention	Attention to wound, chewing, licking, scratching, scratching alongside wall	3.97% (15)
	*Note: 5.82% (22) codes dropped*	

Categories of pain signs as reported by owners (*n* = 51 dogs) and the frequency of the coded pain signs per category as percentage of the total number of codes (*n* = 378 codes).

Seven of these identified pain sign descriptors and five pain signs taken from the Glasgow Composite Measure Pain Scale -Short form [20] were added to complete the list of owner-derived pain signs, resulting in 18 categories and 100 pain signs. (S2 Table lists the owner-derived pain signs and the additions per source).

### A basis for an acute pain scoring instrument

From the list of 18 categories created as described in the section ‘Qualitative analysis of free text questionnaire entries and veterinarians’ descriptions’, four categories were dropped: ‘elimination change’ and ‘intake change’, as corresponding behaviours may reflect other causes than a dog’s pain experience; ‘normal routine’ and ‘sleepiness/tiredness’, as these were deemed to be covered by already formed categories. The categories ‘alertness and stress’ and ‘fear and insecurity’, as well as ‘muscle tension’” and ‘position and resting difficulty’ were combined, following the assumption that the grouped descriptors indicate similar behaviours. Three categories referring to impaired movement - ‘mobility limited’, ‘movement impaired’ and ‘movement lacking’ – were combined, based on the same assumption of indicating similar behaviour. Changes in greeting behaviour were investigated separately according to emphasized importance in owner reports and literature [26]. Considering the different levels in pain severity indicated by vocalisation types [20], the category ‘vocalisation change’ was split in two. The adjustments resulted in a list of 11 categories, as shown in Table 4. A question investigating occurrence or absence of examples resembling pain-induced behavioural changes, was formulated for each category. The last category ‘attention to the wound area’ was supplemented with a sub question investigating distractibility when attentive to the potentially painful area, completing the basis for the development of an owner-directed dog acute pain scoring instrument in the future.

**Table 4:** Proposed dog-owner-directed questions requiring validation.

Category	Question
Aggression	Is your dog showing uncharacteristic aggression behaviours? Examples are: growling or snapping. Some dogs may guard the wound or painful area and show defensive behaviours particularly when someone is near that area.
Fear and stress	In the home environment, is your dog more fearful or stressed than before onset of the condition or treatment? Examples are: hiding, panicking, shaking, low tail carriage, appearing confused or unsettled, looking around a lot, breathing rapidly, licking lips repeatedly, outside of eating or drinking moments.
Movement impairment	Is your dog moving carefully or with difficulty? Examples are: careful movements, stiff and/or slow movement, pausing often while moving, reluctance to walk, not putting weight on one or more limbs, not able to get up, lameness, lifting a leg, dragging a leg, and/or being unsteady when moving, lying down again quickly.
Greeting	Is your dog greeting you less? Examples are: coming to greet you less when you enter the home, or when you enter the room where the dog is.
Muscle tension and restlessness	Does your dog appear tense, rigid or uncomfortable? Examples are: tense muscles in the face or body, a rigid position, changing position often, getting up often while resting, showing unnatural postures, e.g., a tucked belly line or a hunched back.
Owner avoidance	Is your dog avoiding you or other household members? Examples are: keeping distance, looking away or avoiding touch such as petting.
Owner-directed	Is your dog directed more at you or other household members? Examples are: monitoring your movement, asking for petting more, staying close more, seeking your attention more.
Quiet and depressed	Is your dog more quiet and calm than normal? Examples are: being lethargic, appearing depressed, appearing sad such as through showing sad eyes or walking with the head held downward.
Vocalisation changes	Is your dog groaning or whimpering?
Intense vocalisation	Is your dog screaming?
Wound attention	Is your dog attentive to a wound/ painful area? Examples are: turning its head toward the area, chewing or licking the area, rubbing or scratching the area.
Wound attention – distractibility	If your dog is attentive to the wound area, is it distractable? Examples are: stopping the attentiveness to the wound area if you call the dog’s name, or if something happens in the home or outdoors.

Dog owner – directed questions, drafted based on categorized behavioural changes derived from the analysis of owner-provided survey-entries.

## Discussion

In preparation for the development of a dog owner-directed acute pain scale, this study identified pain-related behavioural changes, that owners recognized in the first week after their dog’s clinical discharge, as well as the wording used to describe these. To this end, owners of 51 dogs that were treated as inpatients at the Small Animal Clinic of Utrecht University, were surveyed online, and the dogs in question were assessed for their level of pain as part of a veterinary video analysis. Potential participants were informed upfront about the study’s focus on pain recognition by untrained dog owners, which is why two dog owners who were veterinarians, withdrew from participation, in order to avoid skewing the results. The sex distribution among the dogs included was almost equal, as was the proportion of neutered animals among the sexes. The age of the participating dogs ranged from 4 months to 12 years, and the corresponding treatments covered a broad surgical spectrum, from skin tumour removal and castration to total hip replacement and hypophysectomy. This diverse sample consequently included dogs with highly different conditions and prerequisites, but for which the same objective applied, namely freedom from pain in the postoperative period.

Behavioural changes documented by dog owners within the first three days after discharge regarded changes predominantly in walking (72.1%), playing with an object (70.5%), playing with the owner (68.9%), urination (65.6%), explorative behaviour (62.3%), sleeping (60.7%) and eating (49.2%). An analysis of the accompanying free text entries revealed that with ‘changed’, owners indicated a decrease in eating, playing, and explorative behaviour, whereas sleeping duration was perceived as increased. In this context, it remains unclear to which extent the sleeping duration was influenced by side effects of medication and whether owners were able to distinguish between genuine sleep and feigned sleep [44]. Described changes in urination regarded an increased duration of the urine flow and lighter urine colour; both changes could be related to prior intravenous fluid administration during the inpatient hospital stay [43]. All dog owner-documented behavioural changes except changes in urination, were significantly associated with a higher owner-estimated pain score. These findings indicate, in line with results of previous studies, that owners may find changes in movement-related behaviours more obvious resembling signs of (severe) pain than subtle behavioural changes [27,45,46].

More importantly, the results reflect the possible expression of pain in form of deviations from routine behaviours – signs of pain that may only become apparent in the home environment expressed towards or in the presence of familiar persons. These more individual behavioural changes as well come into play in the context of applying dog owner-directed chronic pain scales [23,26,47]. Studies on owners of dogs suffering from chronic pain reveal that dog owners are able to recognize signs of pain, but run the risk of contextualizing them [35]. Yet, competency in qualitatively assessing a dog’s pain was proven in the Free Choice Profiling methodology mentioned in the introduction [28]. This indicates that using owner vocabulary to describe the observations of their dogs, may prove effective for a dog pain assessment instrument, that can be easily applied and is tailored to the home environment [21,48].

Our study has several limitations. Accounting for only 51 dogs, the sample size was small and the sample collection was non-random. Only owners of dogs scheduled for elective surgery at a university clinic were invited to participate. In order to cover a broad spectrum of patient and treatment characteristics, no restrictions were imposed with regard to patient characteristics such as age, pre- or coexisting conditions, sex, or type of treatment. Despite the diverse sample, the generalizability of the results is limited due to the small sample size and should be verified in a larger study with a participant number sufficient to allow for comparison between patient groups. The participating dog owners were informed about the purpose of the study, and, obviously, about the procedure their dog underwent. A certain observer bias can therefore be assumed, depending, among other things, on owner-deemed level of pain of the dog’s treatment, which in turn could be affected by their personal experiences [26]. In addition, the participants may have focused on possible changes in behaviour to a greater extent than they would have done without participating in a study focused on pain sign recognition. Correspondingly, it should also be noted that the survey was published and completed in Dutch. Despite great care being taken in translating it into English, this means that the objective of capturing colloquial expressions used by dog owners, could only be achieved to a limited extent. While clear descriptions, understandable to laypeople, could be extracted and translated, idioms that are only commonly used in Dutch may have been lost. The influence of language and cultural background will be particularly important in the future creation of a dog’s acute pain scale, and it must be noted that, based on participant wording in one language, a pain scale may only be validated for this specific language [49]. Moreover, the pain experienced by the dogs in question at the time of documentation was not verified by physical examination. Alternatively, a veterinary video analysis served to verify the pain assessment provided by the dog owners and thus to substantiate that the documented behavioural changes were pain-related [50,51]. Yet, as owners selected when to video record their dogs, our approach may not have captured all situations reflective of a dog’s pain experience. Asking the participants to record behaviours they deemed indicative of their dog’s pain, followed logical from our study objective. We found moderate to high correlation between video assessment of the three veterinarians. The assessors may have been biased by personal and professional experiences [52], as well as by the fact that multiple videos referred to the same dogs, which may have led to a recognition effect. In addition, the veterinarians conducting the assessment were not informed about the patient characteristics or the types of procedures performed, but they were informed that the dogs included had undergone clinical treatment, which may have influenced their evaluation. At the time of the study, almost half of the dogs included were receiving gabapentin as pain medication, which can have a sedative effect, especially at the start of treatment [53,54]. Other side effects that have been reported after gabapentin administration in dogs include vomiting, mild ataxia and restlessness [55]. While vomiting was not observed among the recordings of participating dogs, atactic movements could potentially have been confused with careful movement due to pain; especially in consideration of the limited duration of the videos the assessors were provided with to base their evaluation on. With restlessness being also a possible pain sign [56], it may have been interpreted as such, particularly in the study context. Statistically, for two of the evaluating veterinarians, a European diplomate in Veterinary Anesthesia & Analgesia (ECVAA) and a European diplomate of Veterinarian Emergency and Critical Care (ECVECC), a significant correlation between pain scoring results and gabapentin medication was found, in that gabapentin administration increased the chance for a pain score of over 5 out of 10. This did not apply for the pain scoring results of the non-specialized veterinarian and the participating dog owners. It should be noted that the dogs receiving gabapentin predominantly underwent surgeries that are classified as more painful than those for which the postoperative analgesic protocol did not include gabapentin (see Table 1 for patient and treatment characteristics) [57]. A recent study investigating qualitative behavioural assessment of dogs with acute pain [28], revealed that signs of stress in dogs were interpreted as signs of pain more often by veterinary students than by veterinarians specialized in analgesia. Insufficient differentiation between behavioural changes attributable to pain and those indicative of other forms of discomfort or sedation may therefore indicate a lack of knowledge in this field. This may affect not only students but also veterinarians who have not undergone specialised training, and could be improved by more comprehensive treatment of behavioural medicine aspects in the veterinary curriculum [58].

The extent to which gabapentin medication specifically influences the expression of pain in dogs and its interpretation, should be further investigated in a study including dogs undergoing the same surgical procedure, that are subsequently treated with either gabapentin or an alternative analgesic. Mean owner-estimated pain level of their dog, expressed as a score ranging from 0–10 on a numerical scale, was 3.4 ± 2.7, differing to a small extend from the height of veterinarian’s scores, referring to 3.9 ± 1.8, 4.2 ± 2.3 and 4.6 ± 2.9, respectively. Regarding the pain score level, a similar pattern was found when analysing subdivisions of the overall sample: for dogs that underwent orthopaedic surgery, the mean owner-provided score was 4.1 ± 2.8, while the veterinarians scored: 4.3 ± 2.2, 5.8 ± 2.4 and 4.3 ± 1.5; and for dogs undergoing soft tissue surgery, the mean owner-estimated pain score was 2.8 ± 2.7 (0−8, n = 32) and those estimated by the veterinarians accounted for 3.7 ± 2.01, 4.2 ± 2.7 and 3.8 ± 3.1. However, correlation between pain scoring results of veterinarians and dog owners was weak for the overall sample (r = −0.1, 0.2, 0.4, p = 0.6, 0.3, 0.2), dogs undergoing orthopaedic surgery (r = 0, r = 0.1 and r = 0.3, all p > 0.05) and dogs undergoing medical treatment or soft tissue surgery (4r = 0, r = 0.1 and r = 0.2, all p > 0.5). The analysis showed a pattern whereby dogs that underwent orthopaedic surgery were rated as more painful by dog owners and veterinarians than those that underwent soft tissue surgery. This may reflect the actual level of pain experienced by the dogs in question, in line with the fact that most orthopaedic procedures are considered to be more painful than soft tissue operations [57]. It is also possible that dog owners equate movement restrictions, which may be more obvious in orthopaedic conditions due to bandages and obvious limping, with an increased level of pain. Finally, it is imaginable that veterinarians were biased by their knowledge of the potentially increased level of pain of orthopaedic procedures. A study in which veterinarians assessed the pain sensitivity of certain dog breeds showed that professional bias can play a role in the assessment of pain [52]. A weak correlation between dog owner and veterinarian estimated pain scores may also be due to the measurement instrument used, a Likert scale. A numerical scale is likely to exhibit similar weaknesses when used by untrained users, such as dog owners, as a visual analogue scale. A visual analogue scale was shown to have poor validity in a study on the assessment of chronic pain by dog owners [59]. This means that if dog owners perceive behavioural changes but do not recognize them as pain-induced, this may lead to a falsely low score on a numerical scale or a visual analogue scale.

In course of our study, we asked owners to submit videos of their dogs showing behavioural changes that made them think their dog was in pain. These specific instructions may have caused a bias in the dog behaviours that owners captured on video. Generally, it is important to note that our study set up did not allow for the identification of more subtle pain signs that owners may not yet recognize in their dogs. This approach refers to our aim to identify pain related behavioural changes, displayed by dogs in the home environment, that are identified as pain related by their owners, and capture the vocabulary owners use for describing pain in their dogs.

Despite these limitations of our study and the need for further studying owner-reported acute pain signs in the home environment, we present an outcome from pain assessments in the home environment with wording used by owners and thus recognisable to them. The identified behavioural categories may provide a foundation for developing a dog owner-directed acute pain scoring system for at home use. To contribute to such a foundation, we drafted dog owner-directed questions based on our study results, taking into account the value of the established dog acute pain scales available for the clinical setting as an environment [20,42,60,61] and incorporating dog owners’ assessment characteristics. This possible foundation for the development of an owner-directed dog’s acute pain scale in the future consists of eleven questions. Each question is illustrated by examples referring to the wording used by owners to describe possibly pain-induced behaviour in their dogs, facilitating the recognition and description of pain-related behavioural changes by dog owners and thus also with a future potential to facilitate the communication between dog owners and the veterinary profession.

## Supporting information

S1 File Questionnaire acute pain sign recognition and video recording instructions. (DOCX)

S2 File Influence of Gabapentin administration on pain scoring results. (DOCX)

S1 Table Characteristics of participating dogs. (DOCX)

S2 Table Dog owner mentioned pain signs, veterinary descriptors and pain scale signs. (DOCX)

S3 Table Correlation between pain scoring results of dog owners and veterinarians. (DOCX)
