# Supplementary material for: Acute pain sign recognition by dog owners in a home setting
Source: PLoS One. 2026 Apr 15;21(4):e0345418. doi: 10.1371/journal.pone.0345418 (PMC13082587; doi:10.1371/journal.pone.0345418)
Supplement: S1 File — (DOCX) [file pone.0345418.s001.docx]

**S1 File. Questionnaire Acute pain sign recognition and video instructions.**

1) How would you describe the behaviour of your dog in three words?

2) How would you describe the posture of your dog in three words?

3) How would you describe the facial expression of your dog in three words?

In this (next) section, please indicate whether you see any change in your dog's behaviour presently (as compared to before your visit to the faculty clinic). Please indicate whether a behaviour is unchanged or changed. If the behaviour has changed, would you additionally please indicate, how it changed, in the text box below (in your own words).

4) Walking: unchanged/ changed

5) Sleeping: unchanged/ changed

6) Eating: unchanged/ changed

7)Urination and defecation: unchanged/ changed

8) Playing with a toy: unchanged/ changed

9) Interaction with you through playing: unchanged/ changed

10) Interaction with you through petting: unchanged/ changed

11) Greeting you when you enter the house / the room in which the dog is: unchanged/ changed

12) Resting near you: unchanged/ changed

13) Exploration - alertness to you or the environment in general: unchanged/ changed

14) Is your dog painful (1 = not at all likely, 2 = unlikely, 3 = don’t know, 4 = likely, 5 = very likely)?

15) How painful is your dog? (0 = no pain, 10 = pain could not be worse):

16) What is telling you that your dog is in pain?

17) What is telling you that your dog is not in pain?

**Video recording Instructions**

Please upload a video of your dog - showing clear signs you see that your dog is in pain.

Details on making the video:

- Please avoid to show other people, private objects (such as bank card, agenda, ID card).
- A duration of 1-2 min is ideal, but please record the entire behavioural sequence you feel is important, even if it is longer. Image: Make sure your dog's entire body is in the picture.
