# Supplementary material for: Acute pain sign recognition by dog owners in a home setting
Source: PLoS One. 2026 Apr 15;21(4):e0345418. doi: 10.1371/journal.pone.0345418 (PMC13082587; doi:10.1371/journal.pone.0345418)
Supplement: S2 File — (DOCX) [file pone.0345418.s002.docx]

**S2 File. Influence of Gabapentin administration on pain scoring results.**

**Table: Relationship between pain scores and gabapentin administration**

| **Painscore** | **Coefficient b** | **p-value** | **Odds ratio (OR)** |
| --- | --- | --- | --- |
| Owner H | -0.21 | 0.717 | 0.81 |
| Owner L | 0.21 | 0.717 | 1.23 |
| Vet A1 H | 0.98 | **0.048** | **2.67** |
| Vet A1 L | -0.98 | **0.048** | **0.38** |
| Vet S1 H | 0.58 | 0.236 | 1.78 |
| Vet S1 L | -0.47 | 0.331 | 0.63 |
| Vet C1 H | 1.46 | **0.007** | **4.29** |
| Vet C1 L | -1.64 | **0.003** | **0.19** |

Analysis of the relationship between pain scoring results (Painscore 10-5 = H (‘High’), 0-4 = L (‘Low’)) of dog owners and veterinarians and gabapentin medication of the respective dogs, tested with logistic regression analysis, with gabapentin (G0/G1 = did not receive gabapentin as an analgesic/ received gabapentin as an analgesic) as independent variable.

A potential effect of gabapentin medication on the evaluation of the dog's pain level was examined using logistic regression analysis. The European diplomate in Veterinary Anesthesia & Analgesia (ECVAA) was significantly (p = 0.048) more likely (OR = 2.67) to score pain with 5 out of 10 or higher in dogs that received gabapentin and slightly less likely (OR = 0.38) to apply a pain score lower than 5. A similar result was revealed for the European diplomate of Veterinarian Emergency and Critical Care (ECVECC), in this case the chance that a pain score of 5 or higher was applied increased more than four times (p = 0.007, OR = 4.29) when the specific dog received gabapentin as an analgesic; the chance for scoring pain lower than 5 was slightly decreased in dogs treated with gabapentin (p = 0.003, OR = 0.19). No significant effects of the dog having received gabapentin as an analgesic was found on the pain scoring results of the non-specialized veterinarian and the dog owners.
