# Supplementary material for: Acute pain sign recognition by dog owners in a home setting
Source: PLoS One. 2026 Apr 15;21(4):e0345418. doi: 10.1371/journal.pone.0345418 (PMC13082587; doi:10.1371/journal.pone.0345418)
Supplement: S1 Table — (DOCX) [file pone.0345418.s003.docx]

**S1 Table. Characteristics of participating dogs.**

| **Dog characteristic** |  |  | **Number of dogs** |
| --- | --- | --- | --- |
| Dogs’ age categories |  | 0 – 1 | 15 |
|  |  | 2 – 6 | 15 |
|  |  | 7 + | 21 |
| Dogs’ sex and status |  | Female – intact | 11 |
|  |  | Female – neutered | 13 |
|  |  | Male – intact | 19 |
|  |  | Male – neutered | 8 |
| Dogs’ treatments |  | Orthopaedic surgery | 20 |
|  |  | Soft tissue surgery | 29 |
|  |  | Other treatment | 2 |
| Analgesic medication for the dog at home |  | NSAIDs | 36 |
|  |  | Gabapentin | 23 |
|  |  | Paracetamol | 22 |
|  |  | Combinations | 23 |

Characteristics of the *n* = 51 dogs’ age, sex and neutering status, their hospital treatment and analgesic treatment at home are presented in absolute numbers.
