# Supplementary material for: Acute pain sign recognition by dog owners in a home setting
Source: PLoS One. 2026 Apr 15;21(4):e0345418. doi: 10.1371/journal.pone.0345418 (PMC13082587; doi:10.1371/journal.pone.0345418)
Supplement: S2 Table — (DOCX) [file pone.0345418.s004.docx]

**S2 Table. Dog owner mentioned pain signs, veterinary descriptors and pain scale signs.**

| **Category** | **Pain signs mentioned by N=51 dog owners and coded with NVivo^TM^ software** | **Veterinarian descriptors** | **CSU-CAP** | **Glasgow CMPS-SF** |
| --- | --- | --- | --- | --- |
| Aggression | Aggression to dogs and/or people  Snapping |  |  | Growl  Guard wounded area |
| Alertness and stressfulness | Alert  Confused  Ear movement  Looking around  Stressed | Lip licking  Unsettled |  | Increased respiratory rate |
| Elimination change | Less elimination  Stool in small amounts  Urinating less  Urination inside |  |  |  |
| Fearfulness and insecurity | Fearful after movement  Frightened  Insecure  Panicking  Shaking |  |  |  |
| Intake change | Drinking less  Drinking more  Eating less  Eating with owner |  |  |  |
| Mobility limited | Careful  Getting up difficult  Lying down again quickly  Movement difficult  Moving slowly  Pausing  Reluctance to walk  Walking slowly | Stiff |  |  |
| Movement impairment | Dragging leg  Instable gait  Lame  Lifting wounded leg |  |  |  |
| Movement lacking | No greeting  No jumping  No playing  No walking  Not getting up  Not moving  Sitting still |  |  |  |
| Muscle tension | Narrow mouth  Rigid |  |  |  |
| Normal routine | Acting normal if active  Normal routines |  |  |  |
| Owner avoidance | Avoid being touched  Keeping distance | Looking away |  |  |
| Owner directedness | Alert when owner moves  Asks for petting  Attachment increased  Attention seeking  Interacting  Interaction different  Looking at owner  Staying close |  |  |  |
| Owner greeting | Greeting enthusiastically  Responding enthusiastically  Tail wag |  |  |  |
| Position and resting difficulty | Changing position  Getting up more often  Hunched back  Light sleep  Restless  Restless at night  Sleepless  Slipping away (lying position)  Uncomfortable position  Unnatural posture |  |  |  |
| Quietness and depressedness | Calm  Depressed  Dull  Indifferent  Lethargic  Lying head down  No reaction  Quiet  Sad eyes  Walking head down | Subdued |  |  |
| Sleepiness and tiredness | Sleeping a lot  Sleepy  Tired |  |  |  |
| Vocalisation change or absence | No vocalisation  Vocalising  Whining | Groaning |  | Screaming |
| Wound attention | Attention to wound  Chewing  Licking  Scratching  Scratching alongside wall | Looking at wound area | Difficult to distract | Rubbing of wound area |
| Codes that were dropped | Bright eyes  Crooked tail  Curious  Diarrhoea  Drinking normal  Eating grass  Eating normal  Less tail wagging  Painful paw  Searching-asking  Sedated  Smacking  Stool colour different  Urine amount increased  Urine colour changed  Vomiting  Wanting to play |  |  |  |

Pain signs mentioned by N=51 dog owners, veterinary descriptors (N=3 veterinarians, adding N=7 pain signs not mentioned by dog owners) and pain scale signs not mentioned by these dog owners and veterinarians (N=2 pain scales, namely the Colorado State University Canine Acute Pain Scale (CSU-CAP; Hellyer et al., 2015) and short-form Glasgow Composite Measure Pain Scale (CMPS-SF; Reid et al., 2007), adding N=7 pain signs not mentioned by dog owners or vets).
