# Supplementary material for: Acute pain sign recognition by dog owners in a home setting
Source: PLoS One. 2026 Apr 15;21(4):e0345418. doi: 10.1371/journal.pone.0345418 (PMC13082587; doi:10.1371/journal.pone.0345418)
Supplement: S3 Table — (DOCX) [file pone.0345418.s005.docx]

**S3 Table. Correlation between pain scoring results of dog owners and veterinarians, tested with Pearson.**

|  | | **Owner** | **Dipl. ECVVAA** | **General Veterinarian** | **Dipl. ECVECC** |
| --- | --- | --- | --- | --- | --- |
| **Owner** | **Correlation** | 1.00 | 0.35 | -0.11 | 0.15 |
|  | **p-value** |  | 0.02 | 0.47 | 0.34 |
| **Dipl. ECVVAA** | **Correlation** | 0.35 | 1.00 | 0.61 | 0.67 |
|  | **p-value** | 0.02 |  | <0.001 | <0.001 |
| **General Veterinarian** | **Correlation** | -0.11 | 0.61 | 1.00 | 0.53 |
|  | **p-value** | 0.47 | <0.001 |  | <0.001 |
| **Dipl. ECVECC** | **Correlation** | 0.15 | 0.67 | 0.53 | 1.00. |
|  | **p-value** | 0.34 | <0.001 | <0.001 |  |
